# 

**Published:** 1890-02

**Authors:** 


### Communications.

### AMERICAN ACADEMY OF DENTAL SCIENCE.

The twenty-second annual meeting of the American Academy of Dental Science, was held at Young's Hotel, in Boston, on Wednesday, November 13, 1889.

There was a very good attendance of members present, and the chair was occupied by the President, Dr. Cecil P. Wilson, of Boston. He welcomed the members to the hospitalities of this twenty-second anniversary, and spoke of the pleasures and benefits derived from gatherings like this of earnest professional gentlemen, united together for the public good, and for the promotion of the best interests of their chosen profession, and he congratulated them upon the prosperity and progress of the academy. After listening to the annual reports, and other routine business having been transacted, the following officers were elected for the ensuing year:

President, Dr. F. N. Seabury, of Providence, R. I.
Vice-President, Dr. C. A. Brackett, of Newport, R. I.
Cor. Secretary, Dr. E. N. Harris, of Boston, Mass.
Rec. Secretary, Dr. V. C. Pond, of Boston, Mass.
Treasurer, Dr. E. C. Briggs, of Boston, Mass.
Librarian, Dr. Charles Wilson, of Boston, Mass.

EXECUTIVE COMMITTEE.

Dr. E. H. Smith, of Boston, Dr. Thomas Fillebrown, of Portland, Maine, and Dr. F. E. Baufield, of Boston.

Dr. W. H. Potter, of Boston, was appointed editor of the academy for the ensuing year. Dr. Harris, corresponding secretary of the academy from 1867 to 1877, by urgent solicitation of the members, consented to accept that position again.

Resolutions of respect to the memory of Dr. F. Searle, of Springfield, Dr. S. P. Stearns, of Boston, and Dr. J. W. Smith, of Newport, members of the academy, who departed this life during the past year, were adopted by a rising vote.

The annual address was delivered by Dr. J. T. Codman, of Boston, taking for his subject "Retrospect and Prospect." He spoke of the conditions of life under which professional men lived seventy-five years ago, and gave a very interesting sketch of their surroundings, and a synopsis of the many improvements made in society and in dentistry since that time.

A vote of thanks was given Dr. Codman, for his address and a copy requested for publication.

A vote of thanks was also presented to the retiring president, Dr. Wilson, for the able and impartial manner in which he has presided over the academy during the past two years. At six o'clock the meeting adjourned to partake of the anniversary dinner and banquet, which was an elegant affair. The newly elected president, Dr. Seabury, presided and he thanked the members for the honor conferred upon him and called upon the venerable Dr. Elisha G. Tucker, who was present, and now in his eighty-second year, for some remarks. Dr. Tucker responded and expressed his gratification at having had the pleasure and privilege of attending every annual meeting since the academy was organized, and spoke of the old pioneer dental practitioners of Boston and other parts of our country, who have nearly all passed away. Other pertinent and well-timed remarks were made by many of the members, and everything passed off very happily.

This society was founded in Boston in 1867, and its career has been one of uninterrupted success, and it has accomplished much for the advancement of dental science and for the elevation of the profession.

Monthly meetings are held at the rooms of the Boston Medical Library association, 19 Doylston Place, which are well attended, and are replete with interest. At the last meeting, (held December 4th,) papers were read by Dr. D. W. Fellows, of Portland, upon "Fracture of the Jaws," and by Dr. J. E. Waitt, of Boston, on "A Rapid Method of Inserting and Finishing Contour Fillings without a Matrix."

Each paper was followed by a discussion of the subject presented. Dr. F. G. Eddy, of Providence exhibited a new mouth-piece for saliva ejectors, and Dr. Waitt exhibited a nitrous oxide light for photo-microscopic purposes.

George T. Baker, D. D. S., of Boston, and William S. Sherman, M. D., D. D. S., of Newport, were elected associate members, and W. W. H. Thackston, M. D., D. D. S., of Farmville, Virginia, was elected an honorary member.

EDWARD N. HARRIS, D. D. S.,
Corresponding Secretary,
366 Columbia Avenue, Boston.

### SOME FURTHER NOTES ON THE INVENTION OF PORCELAIN TEETH BY THE LATE CHAS. W. PEALE.

From a manuscript autobiography in the possession of his grand daughter, Miss M. J. Peale, it appears that Chas. W. Peale had spent an abundance of time in making partial and whole sets of artificial teeth of different animal substances, ivory, sea-cows and sea-horse. But they all proved unsatisfactory, owing to their rapid decay and consequent offensiveness. He now sought to find, if possible, some harder and more durable material. Hogs' teeth proved the hardest, but it was difficult to procure them sufficiently large. By cutting down the grinders of the cow he obtained a tooth tolerably suited to his purpose, and made many sets of them. His mode was, " first to form a plate of pure silver to fit the gums perfectly and then to solder a thin plate around each plate that fitted the gums in a perpendicular position. On the front of this plate the teeth were riveted, a single rivet being generally sufficient for each tooth." " To these sets he attached springs invented by his friend, John Dorsey, which were greatly superior to any before used, as they permitted the jaws to open to their full extent and allowed grinding motion also. They were set close to the cheeks without causing any iritation, and had a round head-button on each side of the upper and the lower jaw, to which the springs were attached."

But these materials not proving satisfactory, he determined to make them of porcelain, and prepared for it by studying up the subject of such manufactures. In his labors he was kindly assisted by a Mr. Abraham Miller, who carried on a large pottery in Philadelphia, and who had himself been making experiments in the manufacture of porcelain. In his first attempts, Peale made his porcelain teeth as he did those of other material, with holes for riveting on the plates, but as these soon got out of repair, he determined to try to fuse them to the metal plate, and selected Platinum for the latter, as it would not melt at the heat necessary to fire the porcelain. The glazing and coloring of the teeth gave him much difficulty for a long time, But after two years patient toil and failure, and renewed effort, he finally triumphed over all his difficulties and produced complete and perfect sets of porcelain teeth. This must have been about 1823 or 1824. The patient and persevering efforts of Peale, in this invention, deserve more credit than has been awarded them. We must remember, too, that at the time of these experiments he had passed his eighty-third year of life, a period where most of us who survive to that age, claim exemption from further effort and seek repose. He was at this time, as he writes, in full vigor, his eyesight having returned to such an extent as to enable him to do without glasses. He was also in the zenith of his fame as a patentee, and his time much occupied by calls upon his pencil and brush. Yet his nobility of character is well shown in a remark he makes in the copy of a letter before me, that "the making of porcelain teeth is my present favorite occupation, as by it, *I can give more comfort to my fellow-men*, than by any other work I can undertake." A well deserved compliment to the dental art, and I submit that sufficient justice has not been done to the memory of the man who did so much to popularize the use of artificial teeth. With all the modern appliances that dentistry now has to turn out perfect work in this respect, we are too apt to forget the merits and difficulties of him who originated porcelain teeth. All of us can find the newly discovered land, when some Columbus leads the way—we can even improve on his efforts—but none the less does the discoverer deserve our gratitude and remembrance. It is a singular fact—one that is a source of just pride to the Maryland dentists that our state was the first in the world to establish a Dental College—and from the facts contained in this paper, that which tended more than anything else to extend the usefulness of the art—the invention of Porcelain teeth was also due to the genius and persevering efforts of a son of the same soil—Charles M. Peale.

JNO. R. QUINAN, M. D.

### To Readers and Correspondents.

Communications intended for publication, and exchange journals and papers, should be addressed to the Editor of The American Journal of Dental Science, No. 845 N. Eutaw St. Baltimore, Md. All letters relating to the business of the Journal, or containing cash remittances, to be directed to Messrs. Snowden & Cowman, No. 9 W. Fayette Street, Baltimore, Md. Articles for publication should be received by the Editor at least two weeks previous to the first of the month on which the number in which it is designed for them to appear, is issued.

☞ The American Journal of Dental Science will contain fifty pages of reading matter in each number, making a volume of about Six Hundred pages,

EXCLUSIVE OF ADVERTISEMENTS.

